# Investigation of performances of commercial diesel oxidation catalysts for CO, C_3_H_6_, and NO oxidation

**DOI:** 10.3906/kim-2012-18

**Published:** 2021-06-30

**Authors:** Hande GÜNEŞ, Deniz ŞANLI YILDIZ, Hüseyin Barkın ÖZENER, Gökhan HİSAR, Selmi Erim BOZBAĞ, Can ERKEY

**Affiliations:** 1 Department of Chemical and Biological Engineering, Faculty of Engineering, Koç University, Sarıyer, İstanbul Turkey; 2 Ford Otosan R&D Center, Sancaktepe, İstanbul Turkey

**Keywords:** aftertreatment systems, diesel oxidation catalyst, bimetallic catalyst, platinum, palladium, commercial monolith

## Abstract

Four commercial monolithic diesel oxidation catalysts (DOCs) with two different platinum group metal (PGM) loadings and Pt:Pd ratios of 1:0, 2:1, 3:1 (w/w) were investigated systematically for CO, C_3_H_6_, and NO oxidation, CO-C_3_H_6_ co-oxidation, and CO-C_3_H_6_-NO oxidation reactions via transient activity measurements in a simulated diesel engine exhaust environment. As PGM loading increased, light-off curves shifted to lower temperatures for individual and co-oxidation reactions of CO and C_3_H_6_. CO and C_3_H_6 _were observed to inhibit theoxidation of themselves and each other. Addition of Pd to Pt was found to enhance CO and C_3_H_6_ oxidation performance of the catalysts while the presence and amount of Pd was found to increase the extent of self-inhibition of NO oxidation. NO inhibited CO and C_3_H_6_ oxidation reactions while NO oxidation performance was enhanced in the presence of CO and C_3_H_6_ probably due to the occurrence of reduced Pt and Pd sites during CO and C_3_H_6_ oxidations. The optimum Pt:Pd ratio for individual and co-oxidations of CO, C_3_H_6_, and NO was found to be Pt:Pd = 3:1 (w/w) in the range of experimental conditions investigated in this study.

## 1. Introduction

Aftertreatment systems (ATSs) are used to abate exhaust emissions of diesel vehicles. In many of the currently employed configurations, diesel oxidation catalyst (DOC) is the first unit in the ATS of a heavy-duty diesel vehicle. The primary function of DOC is the oxidation of NO, CO, and unburnt hydrocarbons to NO_2_, CO_2_, and H_2_O. Moreover, the heat generated by highly exothermic oxidation of injected diesel in DOC is utilized for active regeneration of the diesel particulate filter (DPF) which is generally placed after the DOC. NO_2_ resulting from oxidation of NO in DOC is also used as an oxidant for passive regeneration of the DPF. Additionally, the ratio of NO_2_ to NO_x_ influences the rate of NO_x_ reduction in selective catalytic reduction (SCR) unit, which is usually placed after the DPF [1]. Although hydrocarbon oxidation by O_2 _is highly favored in lean exhaust conditions of diesel engines, NO_x_ reduction by hydrocarbons and CO also takes place in the DOC, which is known as hydrocarbon-SCR (HC-SCR) and CO-SCR, where hydrocarbons and CO are oxidized by NO_2_ and NO; yielding NO, N_2_O, N_2_ , H_2_O and CO_2 _[2].

DOCs used in ATSs are usually wash coated on cordierite monoliths with square channels [1]. Platinum group metals (PGMs) are generally used as the active metal in DOCs and Pt is the widely used PGM. Since PGM cost is the major contributor to the ATS cost, numerous studies have been made to decrease the amount of PGM in DOCs [3–12]. The addition of Pd was shown to increase thermal stability and sintering resistance of the catalyst [1,6,7]. There have been many efforts to find the optimum Pt:Pd ratio for maximizing oxidation performance of DOCs while maintaining high durability. Shakya et al. reported the performances of four bimetallic PtPd/Al_2_O_3_ DOCs with 11 g/ft^3^ PGM loading for the oxidation of CO, NO, and various hydrocarbons [8]. CO oxidation activity of the catalyst increased with decreasing Pt:Pd ratio, while unsaturated hydrocarbon oxidation activity of the catalyst firstly increased and then reached a plateau with increasing Pt:Pd ratio. NO oxidation performance monotonically increased with increasing Pt:Pd ratio. Kim et al. reported similar results for the catalytic activity of NO oxidation [9]. In their experiments, catalysts with 50 g/ft^3 ^PGM loading were used. Pt-only catalyst and bimetallic PtPd/Al_2_O_3_ with Pt:Pd ratio of 7:1 (w/w) performed very similarly for NO oxidation. Hazlett et al. showed that Pt was more prone to poisoning by CO and partial oxidation products of C_3_H_6_ compared to Pd [10]. Daneshvar et al. and Kang et al. experimented with several bimetallic DOCs with total PGM loading ranging between 17–27 g/ft^3^ and with different Pt:Pd ratios. They found that the overall optimum Pt:Pd ratio was 1.8:1 (w/w) for maximizing hydrocarbon and CO oxidation [11–13]. 

Another important factor which affects DOC performance for a particular component is promotion and inhibition effect associated with the composition of the exhaust mixture [14]. These effects also depend on the PGM loading and Pt:Pd ratio of the catalysts. Khosravi et al. studied oxidation of C_3_H_6_ and NO along with C_3_H_6_-SCR of NO via transient measurements using two commercial monolithic DOCs with a total PGM loading of 95 g/ft^3^ [15]. One of the monoliths was Pt-based, and the other contained a bimetallic catalyst with Pt:Pd ratio of 4:1 (w/w). Conversion of NO to NO_2_ started after conversion of C_3_H_6_ had reached 100%. They also observed that NO inhibited C_3_H_6_-SCR reactions while C_3_H_6_ promoted them. Similar results were reported for C_3_H_6_-SCR reactions by Watling et al. for a commercial monolithic DOC with 120 g/ft^3^ PGM loading and Pt:Pd ratio of 2:1 (w/w) [16]. Kim et al. also showed the sensitivity of NO oxidation to the presence of hydrocarbons [9] They demonstrated the inhibitory effects of several hydrocarbons on NO oxidation over monolithic DOCs with various Pt:Pd ratios. Shakya et al. observed that the NO_2_/NO_x_ ratio at the outlet decreased in the presence of various hydrocarbons due to HC-SCR reactions over bimetallic DOCs with total PGM loading of 11 g/ft^3^ and Pt:Pd ratios of 1:3, 1:1, 3:1, and 10:1 [8]. Similarly, presence of CO decreased NO_2_/NO_x_ ratio at the outlet due to CO-SCR reactions. Hauff et al. investigated CO and NO oxidations and their influence on the oxidation of each other over a commercial monolithic Pt-based DOC with 130 g/ft^3^ PGM loading [17]. CO oxidation was inhibited by the presence of NO, and NO oxidation started after 100% CO conversion was reached when a Pt-based DOC was used. Maximum NO conversion in the presence of CO was higher than that in NO-only oxidation reactions. The promotion of NO oxidation was explained by the prevention of Pt oxide formation at temperatures providing high CO coverage.

This paper investigates the performances of four monolithic commercial DOCs for the aftertreatment of heavy-duty diesel engines for CO, C_3_H_6_, and NO oxidation, CO-C_3_H_6_ co-oxidation, and CO-C_3_H_6_-NO oxidation with various feed concentrations and gas hourly space velocities (GHSVs) via transient activity measurements in synthetic engine exhaust environment (10% O_2_, 7% CO_2_, 5% H_2_O, and N_2_ as the balance gas). Effect of Pt:Pd ratio and total PGM loading on oxidation performance were investigated. Light-off temperatures were compared for C_3_H_6_ and CO oxidation reactions while maximum conversion and T_20_, temperature at 20% conversion, were compared for NO oxidation reaction to assess catalyst performances. Self-inhibition of the feed gases on oxidation reactions as well as their inhibition/promotion effects on the oxidation of one another in the presence of Pt and Pd were also investigated. 

## 2. Materials and methods

### 2.1. Materials

Pt/Al_2_O_3_ and PtPd/Al_2_O_3_ washcoated honeycomb commercial DOCs (300 cpsi) for the aftertreatment of heavy-duty diesel engines were used in this study. Pt:Pd ratios of the DOCs are given . DOCs were named after their Pt:Pd ratio (w/w). The monometallic Pt DOC was named as PtPd1:0. Bimetallic DOCs with Pt:Pd ratio of 3:1 (w/w) and 2:1 (w/w) were named as PtPd3:1 and PtPd2:1, respectively. Bimetallic DOC with approximately 15% lower total PGM loading and Pt:Pd ratio of 2:1 (w/w) was named as PtPd2:1L. Carbon dioxide, nitrogen, and oxygen (99.998%) were purchased from Air Liquide. Water was deionized before usage (18 mΩ). CO (10%, balance N_2_) gas was purchased from Messer. C_3_H_6_ (49%, balance He) and NO (2%, balance N_2_) gases were purchased from Elite Gaz.

### 2.2. Activity measurements

**Figure 1:**
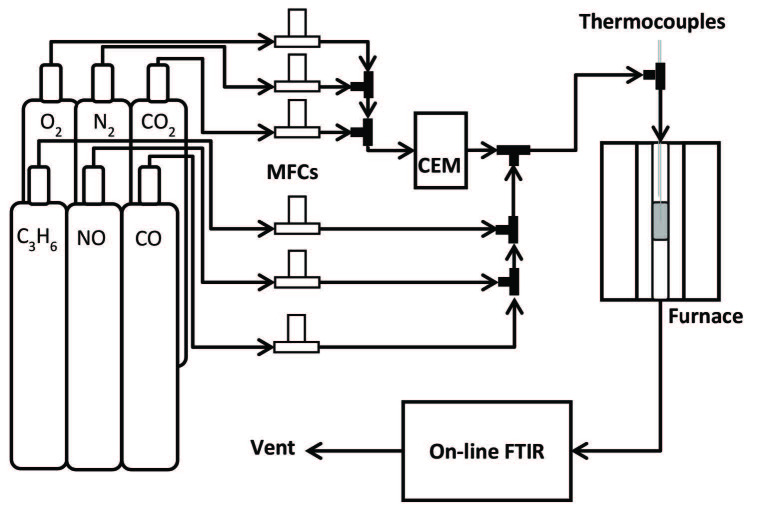
Experimental setup used in activity measurements.

Experimental setup is given in Figure 1. DOC monolithic cylinders with 1.7 cm diameter and 2.5 cm length were drilled out of commercial DOCs which were 13 cm long and had a diameter of 30 cm. The DOC monolithic cylinders were wrapped with insulation to avoid bypass and placed in a stainless-steel tube. Two thermocouples (Omega Inc., CT, USA) were placed at the entrance and at the center of the monoliths. The tube was placed in an electrical furnace (Split Tube Furnace XST-2-0-12-1V2-E28, Thermcraft, NC, USA). Water was delivered by an evaporator-liquid mass flow controller (MFC) system (Controlled evaporator mixer (CEM) system W-202A-220-K, Bronkhors, Nijverheidsstraat, NL t). All other gas flows were controlled via calibrated thermal MFCs (5850E, Brooks Instruments, PA, USA). All lines to and from the reactor furnace were heated above 100 **°** C to avoid water condensation. The concentration of the gases leaving the reactor were measured by an FTIR continuous gas analyzer (MultiGas 2030, MKS Instruments Inc., MA, USA). DOCs were pretreated at 550 **°** C with 10% O_2_, 5% H_2_O, and N_2_ as the balance gas for 5 h. After each run, the catalysts were treated with 10% O_2_ and 90% N_2_ at 450 **°** C for 20 min to clean the surface of the catalyst of any residues. In all experiments, a base feed gas stream composed of 10% O_2_, 5% H_2_O, 7% CO_2_, and N_2_ as the balance gas was used to mimic exhaust gas composition. All experiments were conducted at GHSVs of 75,000 h^–1^ or 100,000 h^–1^. Feed concentrations for CO, C_3_H_6_, and NO ranged between 250–500 ppm, 300–400 ppm, and 140–1000 ppm, respectively. Feed concentrations and GHSVs of individual experiments are summarized in - respectively for PtPd 2:1, PtPd 1:0, PtPd 3:1, and PtPd 2:1L catalysts. Light-off measurements were conducted with a temperature ramp of 10 **°** C/min. The temperature ramp was started after the effluent concentrations of all gases from the reactor were steady. During our experiments, possible byproducts of C_3_H_6_ partial oxidation such as CO, ethylene, formaldehyde, and acetaldehyde were also monitored and no traces of these byproducts were present in the effluent gas stream. 

## 3. Results and discussion

### 3.1. CO oxidation

**Figure 2:**
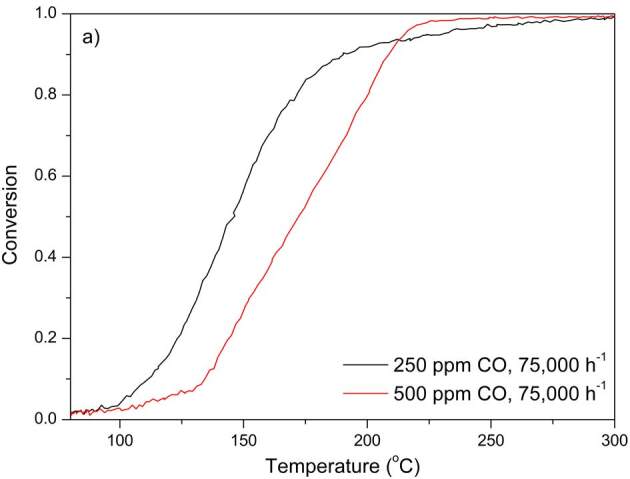

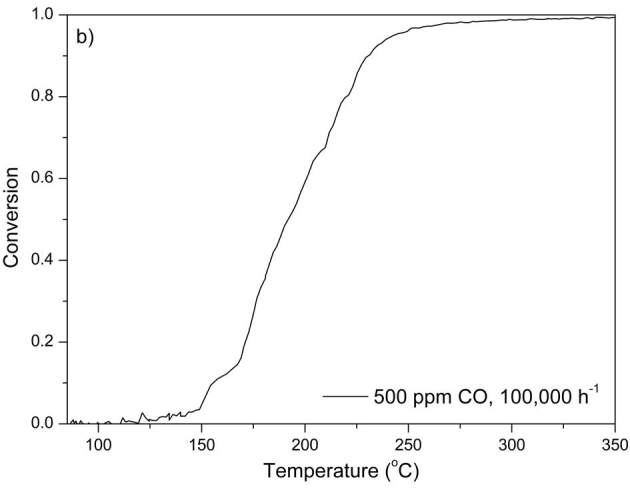

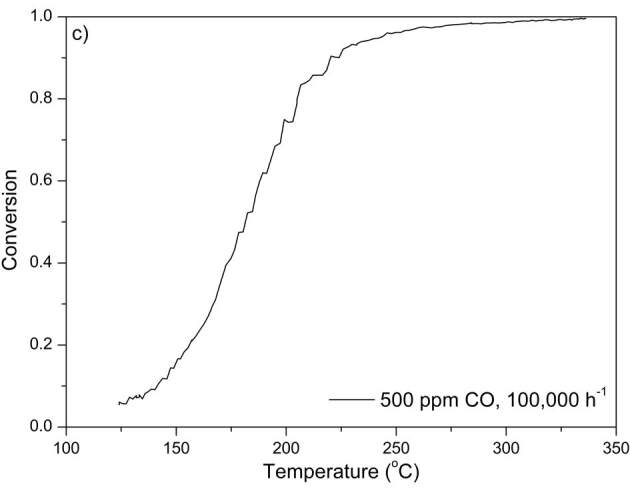

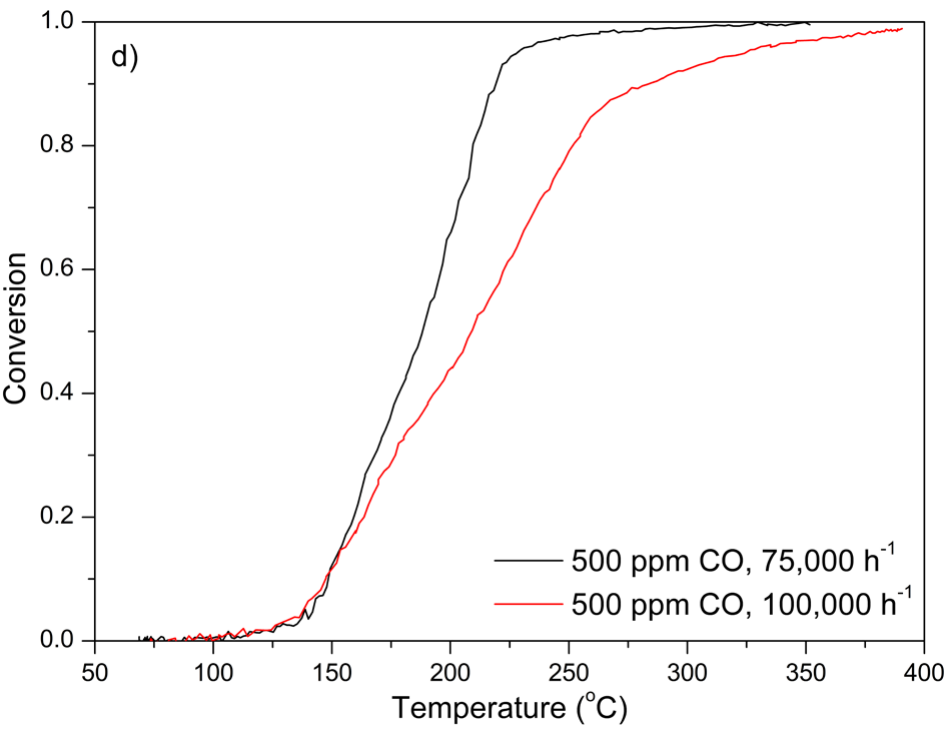
CO oxidation performances of the DOCs. a) PtPd 2:1; b) PtPd 1:0; c) PtPd 3:1; d) PtPd 2:1L. Other gases and their compositions in the feed mixture were 10% O2, 5% H2O, 7% CO2, and N2 as the balance gas.

Light-off curves of CO oxidation for all DOCs are given in Figure 2. Light-off temperatures for CO oxidation are summarized in Tables 2–5. CO oxidation experiments were conducted with two feed CO concentrations for PtPd 2:1. Light-off temperature increased from 146 **°** C to 173 **°** C with increasing feed CO concentration from 250 ppm to 500 ppm, indicating self-inhibition of CO within the concentration range investigated [1,18,19]. 

**Table 1:** Pt:Pd ratios of the commercial DOCs.

Catalyst name	Pt:Pd (w/w)
PtPd 2:1	2:1
PtPd 1:0	1:0
PtPd 3:1	3:1
PtPd 2:1L	2:1

**Table 2:** T50 values for CO and C3H6 in CO, C3H6, CO-C3H6, and CO-C3H6-NO oxidation reactions and T20 values for NO in NO and CO-C3H6-NO oxidation reactions for PtPd2:1 catalyst.

Feed Concentration			GHSV (h–1)	T50 and T20 Values		
CO (ppm)	C3H6 (ppm)	NO (ppm)		CO T50 (°C)	C3H6 T50 (°C)	NO T20 (°C)
250	-	-	75,000	146	-	-
500	-	-	75,000	173	-	-
-	300	-	75,000	-	200	-
-	300	-	100,000	-	215	-
-	-	500	75,000	-	-	342
-	-	1000	75,000	-	-	>400
250	350	-	75,000	171	194	-
500	350	-	75,000	227	228	-
500	350	500	75,000	244	265	339

**Table 3:** T50 values for CO and C3H6 in CO, C3H6, CO-C3H6, and CO-C3H6-NO oxidation reactions and T20 values for NO in NO and CO-C3H6-NO oxidation reactions for PtPd 1:0 catalyst.

Feed concentration			GHSV (h–1)	T50 and T20 Values		
CO (ppm)	C3H6(ppm)	NO (ppm)		CO T50 (°C)	C3H6 T50 (°C)	NO T20 (°C)
500	-	-	100,000	192	-	-
-	350	-	100,000	-	214	-
-	-	500	100,000	-	-	293
-	-	1000	100,000	-	-	300
500	350	-	100,000	216	216	-
500	350	140	100,000	255	259	272

**Table 4:** T50 values for CO and C3H6 in CO, C3H6, CO-C3H6, and CO-C3H6-NO oxidation reactions and T20 values for NO in NO and CO-C3H6-NO oxidation reactions for PtPd3:1 catalyst.

Feed concentration			GHSV (h–1)	T50 and T20 Values		
CO (ppm)	C3H6 (ppm)	NO (ppm)		CO T50 (°C)	C3H6 T50 (°C)	NO T20 (°C)
500	-	-	100,000	182	-	-
-	350	-	75,000	-	134	-
-	350	-	100,000	-	159	-
-	-	180	100,000	-	-	290
500	350	-	100,000	168	173	-
500	350	180	100,000	220	244	257

**Table 5:** T50 values for CO and C3H6 in CO, C3H6, CO-C3H6, and CO-C3H6-NO oxidation reactions and T20 values for NO in NO and CO-C3H6-NO oxidation reactions for PtPd 2:1L catalyst.

Feed concentration			GHSV (h–1)	T50 and T20 values		
CO (ppm)	C3H6 (ppm)	NO (ppm)		CO T50 (°C)	C3H6 T50 (°C)	NO T20 (°C)
500	-	-	75,000	187	-	-
500	-	-	100,000	210	-	-
-	350	-	75,000	-	174	-
-	400	-	100,000	-	209	-
-	-	500	75,000	-	-	277
-	-	1000	75,000	-	-	287
-	-	500	100,000	-	-	290
-	-	1000	100,000	-	-	325
500	350	-	100,000	265	265	-
500	350	500	100,000	255	278	>400

Performances of PtPd 2:1 and PtPd 2:1L for CO oxidation were compared using the light-off data obtained with a feed CO concentration of 500 ppm and at 75,000 h^–1^ GHSV. It was shown that a 15% decrease in total PGM loading resulted in a 14 **°** C increase in CO light-off temperature. CO oxidation performances of PtPd 1:0 and PtPd 3:1 were compared using the light-off data obtained with a feed CO concentration of 500 ppm and at 100,000 h^–1^ GHSV. A 10 **°** C lower light-off temperature was achieved by PtPd 3:1. This indicated that addition of Pd as a secondary metal with constant total PGM amount decreased the CO light-off temperature. This was probably due to lower rate of self-inhibition of CO in the presence of Pd [10,20]. 

### 3.2. C3H6 oxidation

**Figure 3:**
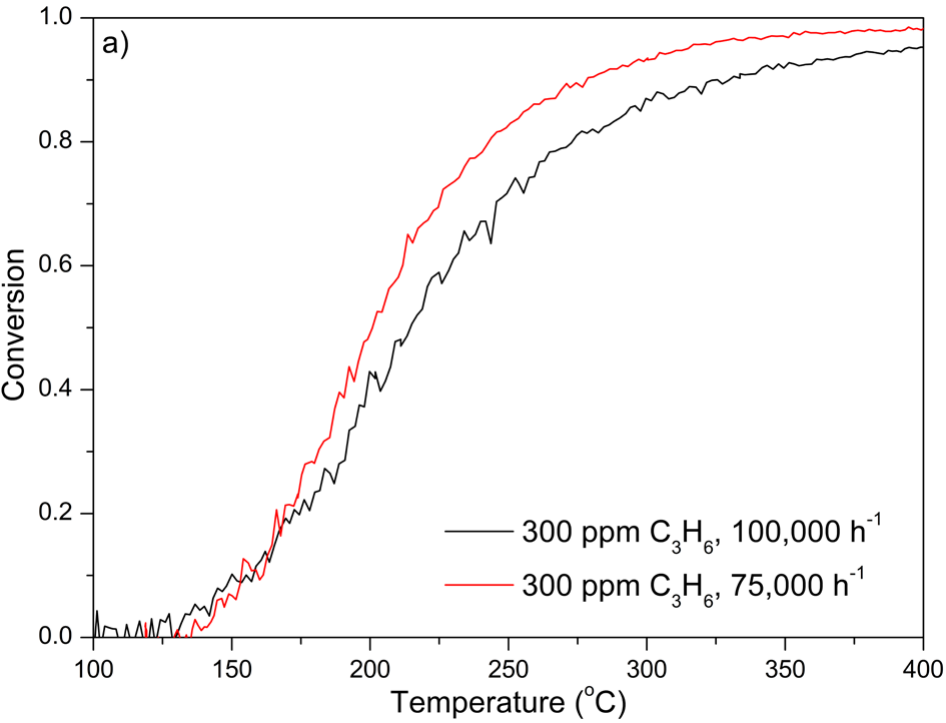

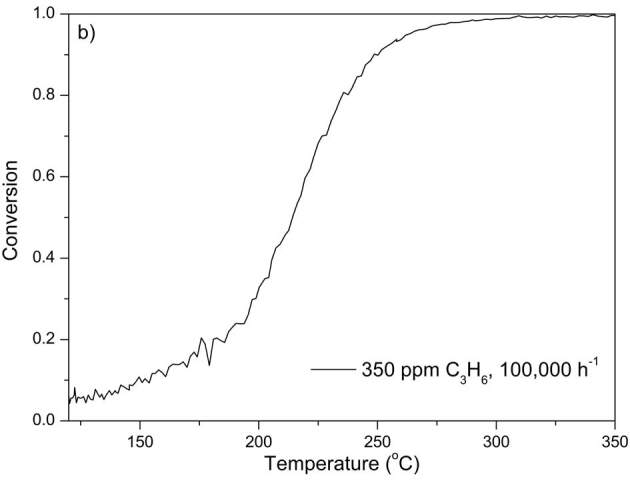

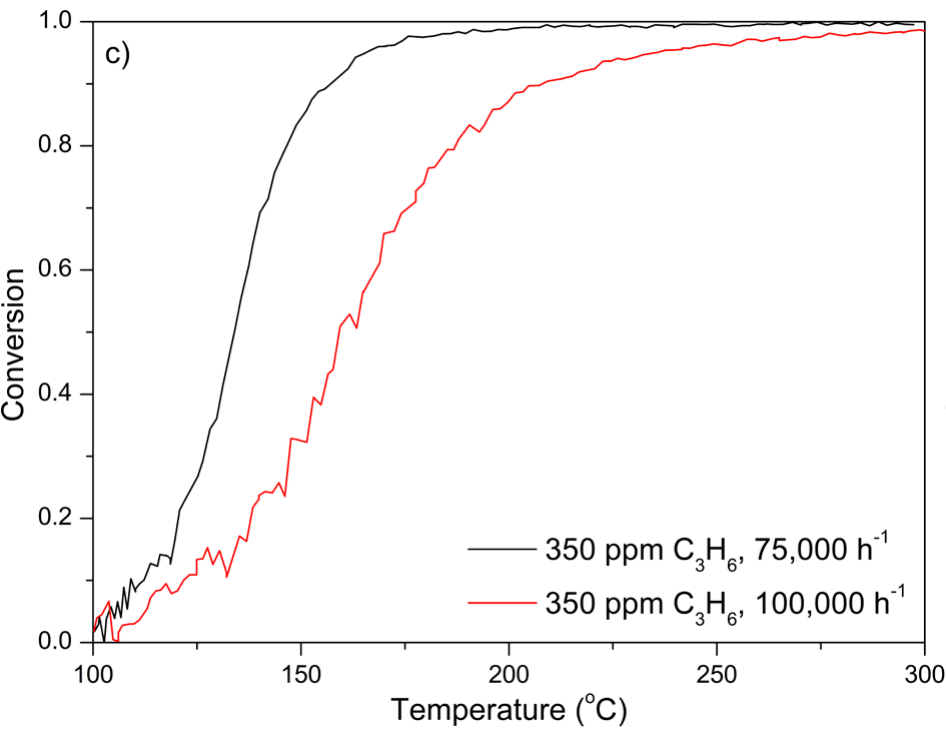

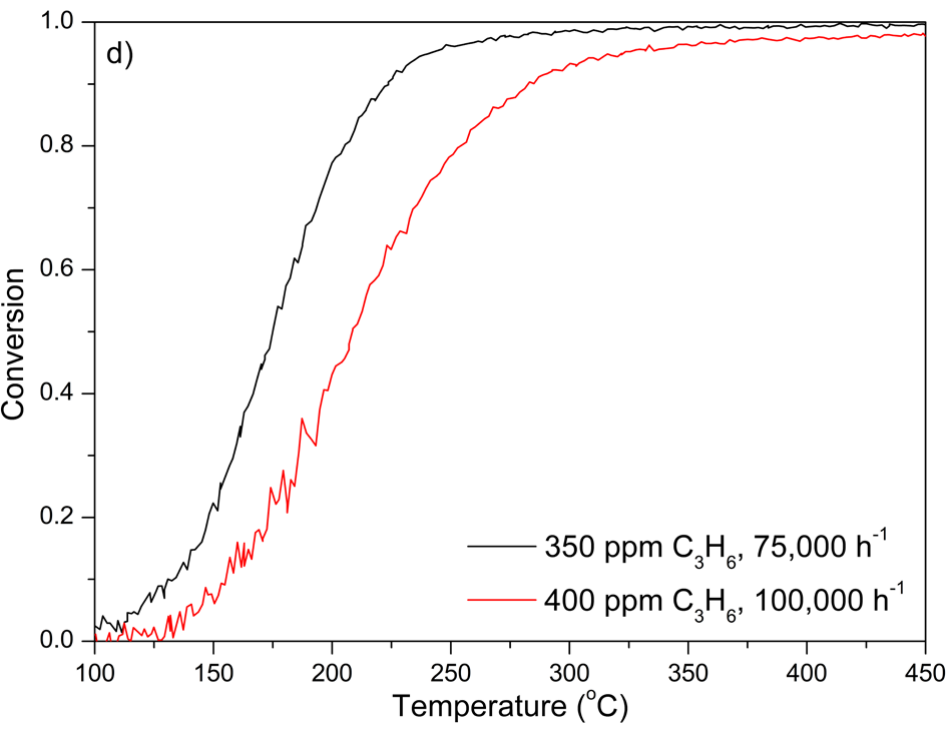
C3H6 oxidation performances of the DOCs. a) PtPd 2:1; b) PtPd 1:0; c) PtPd 3:1; d) PtPd 2:1L. Other gases and their compositions in the feed mixture were 10% O2, 5% H2O, 7% CO2, and N2 as the balance gas.

Light-off curves for C_3_H_6_ oxidation for all DOCs are given in Figure 3 and C_3_H_6 _light-off temperatures are summarized in Tables 2–5. All experiments were conducted with feed C_3_H_6_ concentration ranging between 300–400 ppm and at GHSVs 75,000 h^–1^ and 100,000 h^–1^. 

The lowest C_3_H_6_ light-off temperature was obtained by PtPd 3:1. C_3_H_6 _oxidationperformances of PtPd 2:1 and PtPd 3:1 were compared to investigate the effect of Pd amount of the bimetallic catalysts. PtPd 3:1 resulted in more than 50 **°** C lower C_3_H_6 _light-off temperatures both at 75,000 h^–^^1^ and 100,000 h^–^^1^, compared to PtPd 2:1. The PtPd 1:0 catalyst resulted in a similar light-off temperature with the PtPd 2:1, but the light-off curve was steeper than that of PtPd 2:1. These results indicate an optimum of Pt:Pd ratio of 3:1 for maximum C_3_H_6_ conversion at a particular temperature. Similarly, Dadi et al. compared monometallic Pt, Pd, and bimetallic PtPd DOCs with different Pt:Pd ratios and found that the lowest light-off temperature was obtained by bimetallic DOC with a Pt:Pd molar ratio of 2.3:1 [20].

### 3.3. NO oxidation

**Figure 4:**
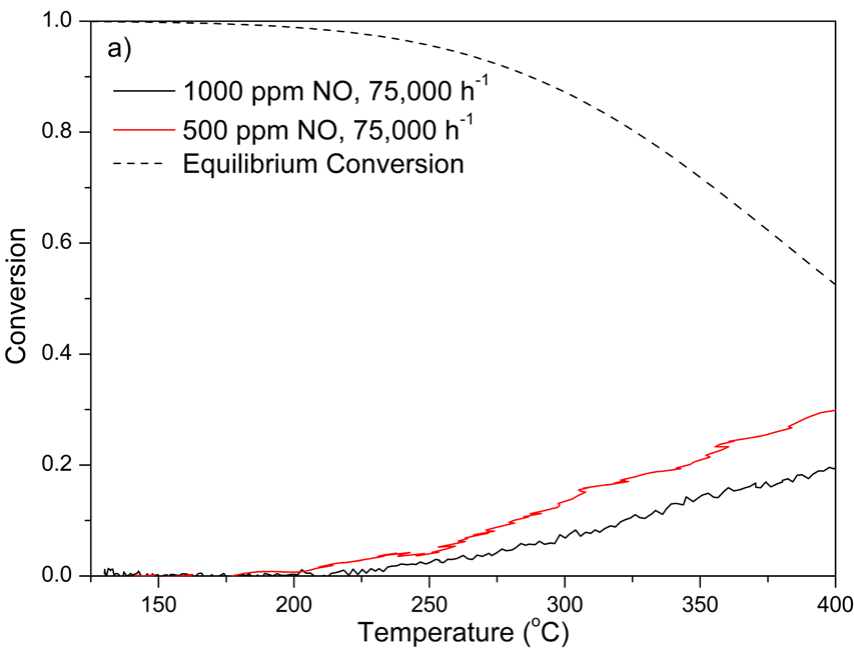

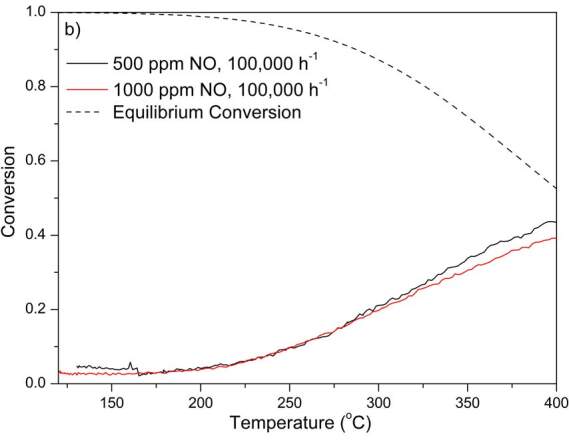

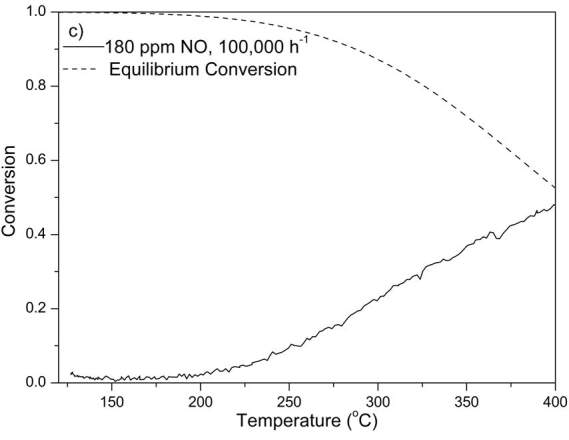

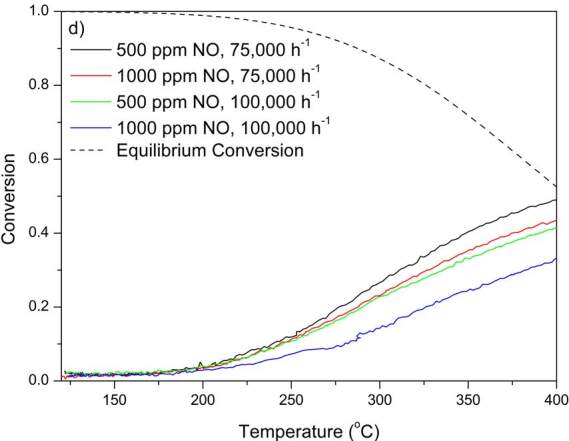
NO oxidation performances of the DOCs. a) PtPd 2:1; b) PtPd 1:0; c) PtPd 3:1; d) PtPd 2:1L. Other gases and their compositions in the feed mixture were 10% O2, 5% H2O, 7% CO2, and N2 as the balance gas.

NO oxidation performances of DOCs are given in Figure 4. T_20 _values are summarized in Tables 2–5. In general, T_20_ values increased with increasing NO feed concentration, which points to the self-inhibition of NO. The self-inhibition was the strongest in PtPd 2:1, which had the highest Pd loading among all catalysts, while it was least pronounced in monometallic PtPd 1:0. PtPd 2:1L performed very similarly with PtPd 1:0 when NO feed was 500 ppm and GHSV was 100,000 h^–1^. When the feed NO concentration was increased to 1000 ppm, a 35 **°** C increase was observed in T_20_ of PtPd 2:1L compared to the 7 **°** C increase in T_20_ of PtPd 1:0. These results suggest that the presence and the amount of Pd has a significant contribution to the self-inhibitory effect of NO on its oxidation. Comparison of the performances of PtPd 2:1 and PtPd 1:0 for NO oxidation showed that although the experiments with PtPt 1:0 were conducted at higher GHSV, T_20_ values were lower than that obtained when PtPd 2:1 was used. As a result, it may be deduced that addition of Pd while keeping total PGM amount constant did not enhance NO oxidation performance of DOC for NO-only oxidation experiments in line with literature [8,9]. 

### 3.4. CO-C3H6 co-oxidation

**Figure 5:**
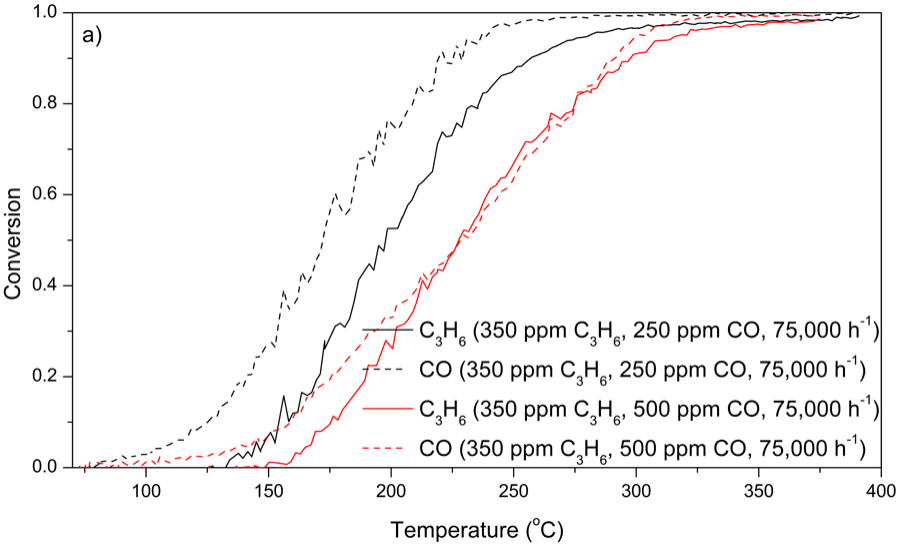

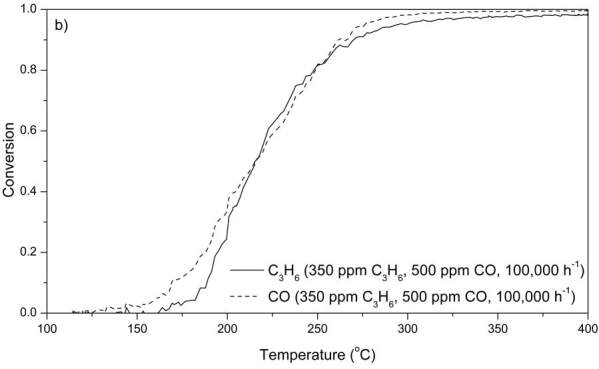

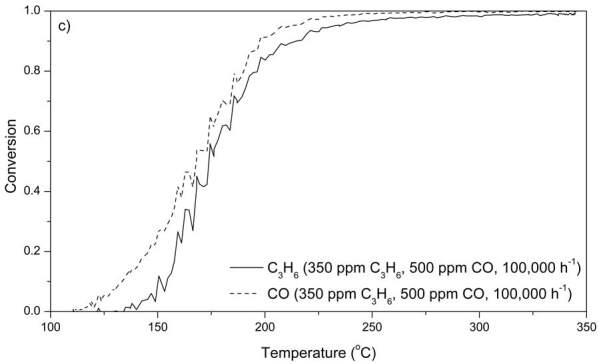

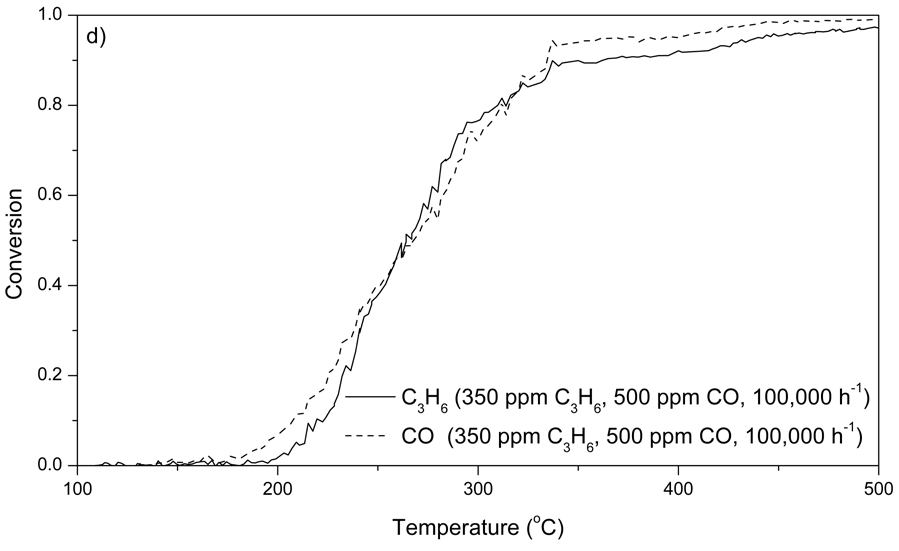
CO-C3H6 co-oxidation performances of the DOCs. a) PtPd 2:1; b) PtPd 1:0; c) PtPd 3:1; d) PtPd 2:1L. Other gases and their compositions in the feed mixture were 10% O2, 5% H2O, 7% CO2, and N2 as the balance gas.

Light-off curves for CO-C_3_H_6_ co-oxidation are given in Figure 5. Light-off temperatures for CO and C_3_H_6_ are listed in Tables 2–5. CO started to oxidize before C_3_H_6 _in all experiments. This was attributed to stronger adsorption of CO on the active sites compared to C_3_H_6 _ [13,21]. Experiments conducted with different CO feed concentrations (250 and 500 ppm) and constant C_3_H_6 _feedconcentration (350 ppm) indicated that the presence of CO inhibits C_3_H_6 _oxidation and the extent of the inhibition increases with increasing CO concentration within the feed concentration range. Comparison of these results with CO-only oxidation experiments indicate that C_3_H_6 _also inhibits CO oxidation reaction. These could be interpreted as competitive oxidation of CO and C_3_H_6 _on the catalyst surface. Inhibition of CO oxidation by the presence of C_3_H_6_ was shown to result from the competition of CO with intermediate species of C_3_H_6_ oxidation for adsorption sites on the surface of Pt/Al_2_O_3_ catalyst [22]. DRIFTS conducted by Hazlett and Epling showed that adsorbed ethylene, adsorbed formaldehyde, and adsorbed acetates were formed as surface intermediates during C_3_H_6_ oxidation, which inhibited both CO and C_3_H_6_ oxidation reactions [21]. Although CO oxidation starts at lower temperatures compared to C_3_H_6_ oxidation, light-off temperatures for CO and C_3_H_6_ were very similar in all experiments conducted with a feed gas mixture containing 500 ppm CO and 350 ppm C_3_H_6_. The lowest light-off temperature for both CO and C_3_H_6_ during co-oxidation were obtained in PtPd3:1, while the highest light-off temperatures were obtained in PtPd 2:1L. These results underline the relation between total metal loading and light-off temperature for CO and C_3_H_6_ oxidation. In this regard, Hauff et al. showed that total amount of metal loading has a direct effect on light-off temperature of C_3_H_6_ and CO during both individual and co-oxidation experiments conducted using monolithic. 

### 3.5. CO-C3H6-NO oxidation

**Figure 6:**
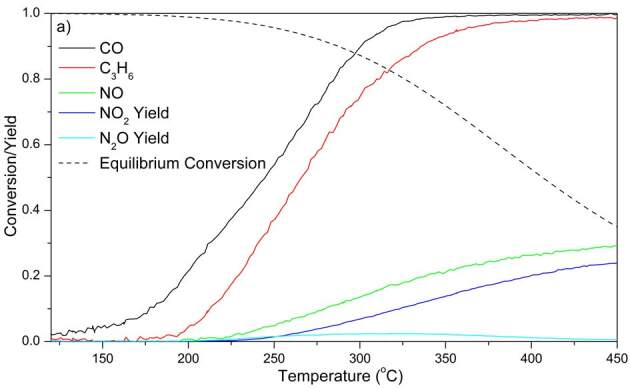

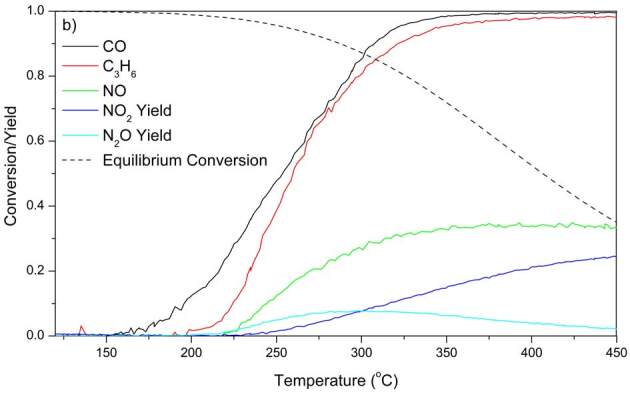

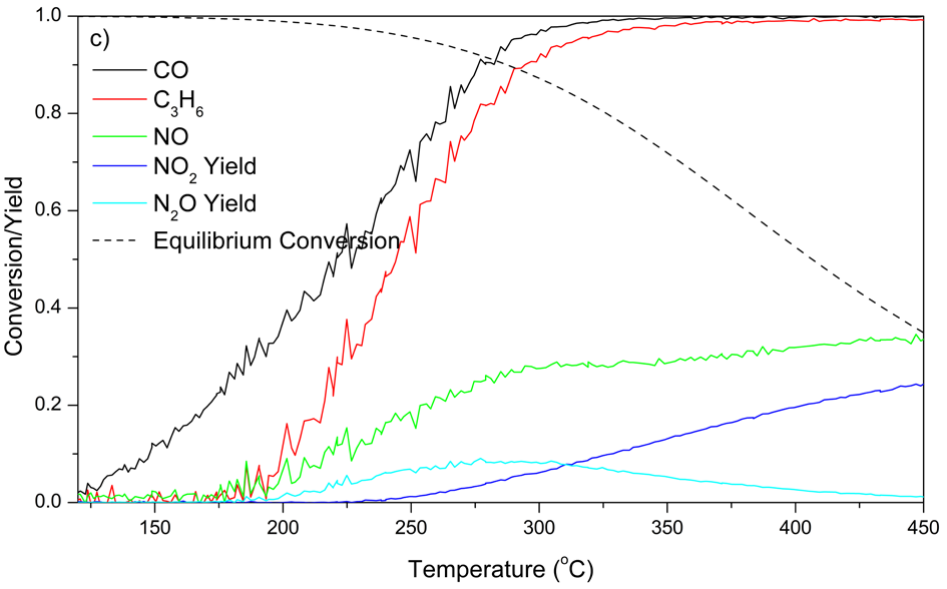

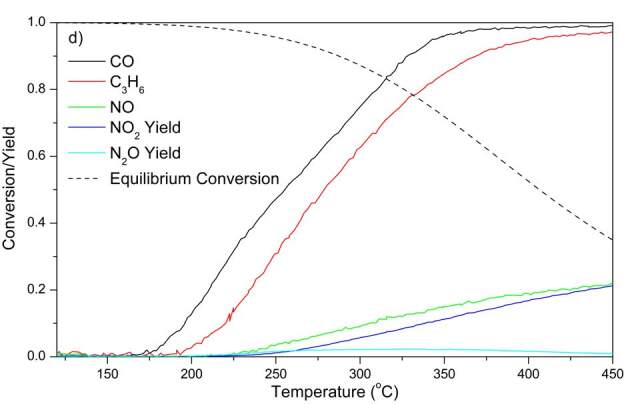
CO-C3H6-NO oxidation performances of DOCs. (a) PtPd 2:1: 350 ppm C3H6, 500 ppm CO, 500 ppm NO at 75,000 h–1 GHSV; (b) PtPd 1:0: 350 ppm C3H6, 500 ppm CO, 140 ppm NO at 100,000 h–1 GHSV; (c) PtPd 3:1: 350 ppm C3H6, 500 ppm CO, 180 ppm NO at 100,000 h–1 GHSV; (d) PtPd 2:1L: 350 ppm C3H6, 500 ppm CO, 500 ppm NO at 100,000 h–1. Other gases and their compositions in the feed mixture were 10% O2, 5% H2O, 7% CO2, and N2 as the balance gas.

Performances of DOCs for CO-C_3_H_6_-NO oxidation are given in Figure 6. T_50_ for CO and C_3_H_6 _and T_20_ for NO are given in Tables 2–5. Addition of NO to feed mixture inhibited both CO and C_3_H_6_ oxidations, as observed by the increase in light-off temperatures of both CO and C_3_H_6_. This points to the inhibition of CO and C_3_H_6_ oxidation reactions by the presence of NO. The lowest light-off temperatures for CO and C_3_H_6_ oxidation during CO-C_3_H_6_-NO oxidation were obtained by PtPd3:1.

At high temperatures, although N_2_O yield is zero, amount of NO converted is higher than NO_2_ yield. The difference between NO conversion and NO_2_ yield was attributed to the formation of N_2_ through C_3_H_6_-SCR and CO-SCR at high temperatures [15,23,24].

Overall C_3_H_6_-SCR reactions taking place on DOCs are given Reactions (1–3) as proposed by Khosravi et al. [15].

C_3_ H_6_+2NO+3.5O_2_→N_2_+3H_2_ O+3CO_2 _(1)

C_3_ H_6_+2NO+4O_2_→N_2_ O+3H_2_ O+3CO_2 _(2)

C3 H_6_+NO_2_+4O_2_→NO+3H_2_ O+3CO_2_ (3)

CO-SCR reactions were mostly pronounced in studies concerning three-way catalysts for aftertreatment of gasoline engine exhaust, however they also take place in DOCs. CO-SCR reactions over noble metal catalysts are given in Reactions (4–6) [25].

2CO+2NO→N_2_+2CO_2 _(4)

CO+2NO→N_2_ O+CO_2 _(5)

CO+N_2_ O→N_2_+CO_2 _(6)

When the extent of NO conversion was compared for NO-only and CO-C_3_H_6_-NO oxidation experiments, it was observed that the maximum NO conversion in the presence of CO and C_3_H_6_ were lower than those in NO-only oxidation reactions except for PtPd2:1; where presence of CO and C_3_H_6 _did not significantly affect NO oxidation profile. However, NO oxidation was considerably hindered by CO and C_3_H_6 _over PtPd 2:1L catalyst. At temperatures lower than 330 **°** C, NO oxidation was enhanced by the presence of CO and C_3_H_6_ for PtPd 1:0 and PtPd 3:1, which was indicated by lower T_20_ values compared to NO-only oxidation. The decrease in T_20_ values can be explained by the conversion of Pt and Pd oxides via C_3_H_6_ and CO at lower temperatures, resulting in reduced Pt and Pd sites, which are more active for NO oxidation [26,27]. The occurrence of C_3_H_6_-SCR and CO-SCR reactions also assisted to the increase of NO conversion at temperatures lower than 300 **°** C. N_2_O yield increased with temperature up to 300 **°** C, and then diminished at higher temperatures. Formation of N_2_O was attributed to C_3_H_6_-SCR and CO-SCR reactions. Higher N_2_O yields were observed by PtPd 1:0 and PtPd 3:1, where NO feed concentrations were lower than that in PtPd 2:1 and PtPd 2:1L. Since NO inhibits C_3_H_6_-SCR [15,28], lower feed NO concentration resulted in higher N_2_O yield and lower NO_2_/NO_x_ ratio.

## 4. Conclusions

Performances of four commercial DOCs with various total PGM loadings and Pt:Pd ratios were investigated for CO, C_3_H_6_ and NO oxidation, CO-C_3_H_6_ co-oxidation, and CO-C_3_H_6_-NO oxidation reactions. Oxidation performances of the DOCs were evaluated by the comparison of light-off temperatures for CO and C_3_H_6_ and maximum conversion and T_20_ values for NO with different gas feed concentrations and GHSVs via transient measurements in synthetic engine exhaust environment (10% O_2_, 7% CO_2_, 5% H_2_O and N_2_ as the balance gas). Effect of PGM loading and Pt:Pd ratio on oxidation performances of these DOCs were investigated and the main conclusions are summarized below:

· It was found that as PGM loading increased, light-off temperature of CO and C_3_H_6_ decreased during individual and co-oxidation reactions. 

· Addition of Pd as a secondary metal decreased CO light-off temperature. The optimum Pt:Pd ratio for maximum CO and C_3_H_6_ performance was found to be Pt:Pd = 3:1 (w/w) for the range of experimental conditions. Presence and amount of Pd was found to increase the extent of self-inhibition of NO oxidation. The lowest T_20_ for NO were obtained by PtPd 1:0 and PtPd 2:1; while PtPd 2:1 was more prone to self-inhibition of NO oxidation compared to PtPd 1:0. 

· CO and C_3_H_6 _were found to inhibit the oxidation of one another. The extent of C_3_H_6_ oxidation inhibition by CO increased with increasing CO feed concentration in the range was investigated. NO inhibited both CO and C_3_H_6_ oxidation reactions during CO-C_3_H_6_-NO oxidation experiments. 

· At temperatures up to 300 ^o^C, NO oxidation over PtPd 1:0 and PtPd 3:1 were enhanced due to C_3_H_6_-SCR and CO-SCR reactions and the occurrence of reduced Pt and Pd sites due to C_3_H_6_ and CO oxidation. 

· In general, addition of Pd enhanced catalytic performance for CO and C_3_H_6 _oxidation. The optimum Pt:Pd ratio for CO, C_3_H_6_ and NO oxidations was found to be 3:1 (w/w) for the range of experimental conditions investigated in this study.
